# Experimental Measurements and Mathematical Modeling of Cytosolic Ca^2+^ Signatures upon Elicitation by Penta-*N*-acetylchitopentaose Oligosaccharides in *Nicotiana tabacum* Cell Cultures

**DOI:** 10.3390/plants2040750

**Published:** 2013-11-27

**Authors:** Kalina Mrozek, Karsten Niehaus, Petra Lutter

**Affiliations:** Department of Proteome and Metabolome Research, Faculty of Biology, Bielefeld University, Universitätsstr. 25, Bielefeld 33615, Germany; E-Mails: kmrozek@cebitec.uni-bielefeld.de (K.M.); kniehaus@cebitec.uni-bielefeld.de (K.N.)

**Keywords:** plant defense, calcium, modeling

## Abstract

Plants have developed sophisticated recognition systems for different kinds of pathogens. Pathogen-associated molecular patterns (PAMPs) can induce various defense mechanisms, e.g., the production of reactive oxygen species (ROS) as an early event. Plant defense reactions are initiated by a signal transduction cascade involving the release of calcium ions (Ca${}^{2+}$) from both external and internal stores to the plant cytoplasm. This work focuses on the analysis of cytosolic Ca${}^{2+}$ signatures, experimentally and theoretically. Cytosolic Ca${}^{2+}$ signals were measured in *Nicotiana tabacum* plant cell cultures after elicitation with penta-*N*-acetylchitopentaose oligosaccharides (Ch5). In order to allow a mathematical simulation of the elicitor-triggered Ca${}^{2+}$ release, the Li and Rinzel model was adapted to the situation in plants. The main features of the Ca${}^{2+}$ response, like the specific shape of the Ca${}^{2+}$ transient and the dose-response relationship, could be reproduced very well. Repeated elicitation of the same cell culture revealed a refractory behavior with respect to the Ca${}^{2+}$ transients for this condition. Detailed analysis of the obtained data resulted in further modifications of the mathematical model, allowing a predictive simulation of Ch5-induced Ca${}^{2+}$ transients. The promising results may contribute to a deeper understanding of the underlying mechanisms governing plant defense.

## 1. Introduction

Plants, as virtually all living organisms, are confronted with pathogens that try to get access to the resources of their hosts. Consequently, plants evolved sophisticated recognition systems in order to sense the pathogen attack and initiate defense mechanisms. From the host point of view, a fast and correct recognition of the pathogen attack is essential. Moreover, the signal transduction machinery of the host has to evaluate incoming signals and generate a decision for or against the onset of the defense mechanisms. This decision is of principle importance to the plant fitness, since plant defense reactions are costly [1]. On the other hand, no defense against a pathogen could be lethal. For this reason, plants evolved a signal transduction machinery that optimizes its defense reactions when confronted with pathogens. One strategy focuses on the recognition of compounds of pathogens (microbial elicitors) on the external face of the host cell [2]. The elicitors, described as pathogen associated molecular patterns (PAMPS), are recognized by pattern recognition receptors (PRRs), and their perception activates the defense machinery of the plant [3]. Parts of the initiation of the plant defense include, e.g., the activation of ion fluxes across the plant cell membrane, rapid changes in protein phosphorylation, production of reactive oxygen species (ROS), along with several more activities, which have been described more extensively in the literature [4,5]. ROS are toxic to the invading pathogen and contribute to the crosslinking of plant cell wall compounds, resulting in a reinforced barrier against infection. Prior to that, the release of calcium ions (Ca${}^{2+}$) from internal compartments is mediated, generating a spike of the cytosolic Ca${}^{2+}$ concentration [5,6,7]. These Ca${}^{2+}$ ions act as a second messenger and are involved in different interactions within the initiation of the plant defense. The cytosolic Ca${}^{2+}$ level is an essential part of the signal transduction network making the decision for or against defense.

A common strategy to measure the cytosolic Ca${}^{2+}$ signatures is to use the Ca${}^{2+}$-sensitive luminescent aequorin system [8]. The detected Ca${}^{2+}$ signals differ in lag period, amplitude and duration, depending on the type of elicitor [9]. Chitin fragments are among some well-known elicitors [10], and Ca${}^{2+}$ measurements using chitooligosaccharides as elicitors are already published, for instance, for parsley, tobacco and soybean cell suspension cultures [7,11,12]. In the course of these experiments, it was observed that a second elicitation with the same elicitor resulted in considerably reduced Ca${}^{2+}$ signals for soybean suspension cells [12].

In contrast to the experimental analysis of Ca${}^{2+}$ signals, only a few mathematical models for plant systems involving Ca${}^{2+}$ transporters are available. Simulations for action potential generation in vascular plant cells [13] and simulations of plant Ca${}^{2+}$ signatures, which are triggered by temperature decrease and the exchange of cytosolic and vacuolar pools [14] are exemplary models. This work focuses on the combination of experimental and mathematical work. For the experimental part, *Nicotiana tabacum* plant cell cultures were treated with penta-*N*-acetylchitopentaose oligosaccharides (Ch5) and the observed Ca${}^{2+}$ signatures were recorded. For the modeling part the minimal version of the De Young-Keizer model [15] for agonist-induced Ca${}^{2+}$ oscillations involving the IP${}_{3}$Rkinetics, reduced by Li and Rinzel [16], was modified in order to achieve a system mimicking the situation in plants. Ca${}^{2+}$ simulations were generated based on the adapted Li and Rinzel model, and different features were analyzed in detail.

## 2. Materials and Methods

### 2.1. Measurement of Cytosolic Calcium Concentration in Tobacco Plant Cell Cultures

Five milliliters of aequorin-expressing tobacco suspension cultures [17], cultivated in the dark, were, three to four days after subcultivation, incubated with 50 µL coelenterazine (1.2 mM in ethanol) on a rotary shaker (orbital rotation, 125 rpm) for 5 h in the dark. Afterwards, 200 µL of these cells were transferred to a cuvette in a single tube luminometer (Berthold Detection Systems, Pforzheim, Germany) and 10 µL of Ch5 of different concentrations in water were added to the sample. Ch5 was obtained from Seikagaku Corporation (Tokyo, Japan). For the refractory measurements, varying quantities of Ch5 concentrations were added to the sample at two different time points, whilst taking into account that the second elicitation had to be done after the first Ca${}^{2+}$ response had ended. All measurements were performed at room temperature, and the elicitor add-on to the sample was done after monitoring a base-line of luminescence. After each measurement, the residual aequorin was completely discharged by adding 200 µL of 1 M CaCl${}_{2}$ in 10% ethanol.

This discharge was used for transforming the luminescent light units into the final Ca${}^{2+}$ concentration, based on the mathematical relationship between the ratio L${}_{i}/$L${}_{max}$ and the final Ca${}^{2+}$ concentration, described in the model B of Allen *et al.* [18]. In this relationship, L${}_{i}$ represents the light intensity at time point i, and the light intensity, L${}_{max}$, is determined by calculating L${}_{tot}$− (L${}_{1}$ + L${}_{2}$ + ...+ L${}_{i}$). L${}_{tot}$ covers the light intensity induced by the complete discharge at the end of the experiment. This formula also includes three constants, which were experimentally fitted by Brini *et al.* [19] ($K_{R}=7.23\times 10^{6}$ M${}^{-1}$, $K_{TR}=120$ and n=2.99).

The tobacco cell cultures used in our work were transformed with a cytoplasmic-targeting apoaequorin expression vector pMAQ2 (A-6793, in the past Molecular Probes). Therefore, the detection focuses on the cytoplasmic localization. The aequorin-system used in this study was already employed for a number of similar experiments [8,12,17]. Due to the size of the aequorin-protein, we cannot rule out that the protein can enter the plants’ nucleus, and part of the signal arises from this location. Recently, Mehlmer *et al.* [20] developed a set of aequorin expression plasmids for the generation of transgenic plant lines to measure calcium levels in different cellular subcompartments. To allow a direct comparison to the already published studies, the pMAQ2 vector-based transformants were used.

### 2.2. Model Description (Adaptation of the Li and Rinzel Model [16])

Cytosolic Ca${}^{2+}$ oscillations of pituitary gonadotrophs can be described and simulated by the Li and Rinzel model. Due to some similar characteristics of the Ca${}^{2+}$ transients in plants, we adapted this approach for further study. Generally, the two-variable model of Li and Rinzel reduces the nine-variable De Young-Keizer model [15] for agonist-induced Ca${}^{2+}$ oscillations, involving IP${}_{3}$ and Ca${}^{2+}$ in the activation process of the receptor channels in the ER. The activation with Ca${}^{2+}$ is defined as the calcium-induced calcium release (CICR). Additionally, a term for a Ca${}^{2+}$ inactivation process of the channels in the ER is included.

In contrast to an animal system, the mechanisms of this described Ca${}^{2+}$ release are not yet affirmed for the situation in plants. The molecular identity of, e.g., IP${}_{3}$-activated channels at the vacuolar and endoplasmic reticulum membranes has not been described so far [21]. Different studies are available that support the existence of ligand-gated channels at these membranes [21,22,23], though. Additionally, there is evidence for the involvement of inositol phosphates in triggering the oxidative burst within the plant defense signal transduction chain [24]. Furthermore, there are published APT-driven Ca${}^{2+}$ pumps that refill the plant Ca${}^{2+}$ stores again [21].

In the following, the Li and Rinzel model will be described more precisely. First of all, it constitutes an analogy to the Hodgkin-Huxley equations for neuronal electrical excitability [25]. This analogy refers to a Ca${}^{2+}$ excitability of the ER membrane, which triggers cytosolic Ca${}^{2+}$ oscillations in response to agonist stimulation, shown, e.g., in pituitary gonadotrophs [26,27]. In the Li and Rinzel model, the cytosolic Ca${}^{2+}$ balance is defined by three different fluxes: a Ca${}^{2+}$ cytosol-inward flux through the IP${}_{3}$ receptor channels, an inward leak flux and an outward Ca${}^{2+}$ pump flux. The Ca${}^{2+}$ pump flux into the ER is defined by the Hill equation term:

$$\begin{array}{c} J_{pump}=\frac{\upsilon_{er}C_{i}^{2}}{k_{er}^{2}+C_{i}^{2}} \end{array}$$

The variable $C_{i}$ (µM) represents the cytosolic Ca${}^{2+}$ concentration. In the Li and Rinzel model, the fraction of free Ca${}^{2+}$ ions in the cytosol and in the ER is involved with scaling the volumes of these compartments. As we could not distinguish between free and bound Ca${}^{2+}$ ions in our experiments, we skipped this scaling parameter in our model. For symbiotic Ca${}^{2+}$ oscillations, it could be shown by Granqvist *et al.* [28] that an increase in buffering capacity in the nucleoplasm would cause a variation in the oscillation patterns of plant root hair cells. However, for the Li and Rinzel model, buffering reduces the Ca${}^{2+}$ fluxes of the cytosol and the ER by several orders of magnitude [26]. Even if we took buffering into account, we would not see the same effects as described by Granqvist *et al.* [28].

The constant $\upsilon_{er}$ (µMs${}^{-1}$) defines the maximal Ca${}^{2+}$ uptake of the pump, and $k_{er}$ (µM) represents the activation constant for this pump. The leak current into the cytosol is defined by a permeability coefficient, p${}_{L}$ (s${}^{-1}$), times the Ca${}^{2+}$ gradient across the membrane of the ER, with $C_{ER}$ (µM) being the Ca${}^{2+}$ concentration of the ER:

$$\begin{array}{c} J_{leak}=c_{1}p_{L}\left(C_{ER}-C_{i}\right) \end{array}$$

The total Ca${}^{2+}$ concentration, C${}_{0}$, in a cell is described as: C${}_{0}$ = C${}_{i}$ + c${}_{1}$C${}_{ER}$, with c${}_{1}$ being the ER/cytoplasm volume ratio. (The volume of the ER is regarded as a subvolume of the cytosolic volume.) The C${}_{ER}$ concentration is then expressed as C${}_{ER}$ = [C${}_{0}$− C${}_{i}$]/c${}_{1}$. Therefore the leak flux can be modified in the following way:

$$\begin{array}{c} J_{leak}=p_{L}\left(C_{0}-\left(1+c_{1}\right)C_{i}\right) \end{array}$$

A last term describes the Ca${}^{2+}$ flux into the cytosol through the IP${}_{3}$ (representing any inositol phosphates in a plant cell as a second messenger) receptor channels situated in the membrane of the ER:

$$\begin{array}{c} J_{chan}=p_{IP_{3}}O\left(IP_{3},Ca^{2+},h\right)\left(C_{0}-\left(1+c_{1}\right)C_{i}\right) \end{array}$$

The constant p${}_{IP_{3}}$ (s${}^{-1}$) describes the maximal permeability of the membrane for the IP${}_{3}$ receptor channels, the variable, IP${}_{3}$ (µM), marks the IP${}_{3}$ concentration, and the expression O(IP${}_{3}$,Ca${}^{2+}$,h) represents the channel open probability at equilibrium with:

$$\begin{array}{c} O={\left(\frac{IP_{3}}{IP_{3}+d_{ip3}}\right)}^{3}{\left(\frac{C_{i}}{C_{i}+d_{act}}\right)}^{3}h^{3} \end{array}$$

This channel open probability has been fitted to experimental data, revealing that a power close to three is the best choice [15,29]. The two dissociation constants, d${}_{ip3}$ (µM) for IP${}_{3}$-binding and d${}_{act}$ (µM) for Ca${}^{2+}$-binding to the IP${}_{3}$ channel, are involved in this expression, as well as a dimensionless variable, *h*, describing channel inactivation. The whole differential equation for the cytosolic Ca${}^{2+}$ balance can be summarized by:

$$\begin{array}{c} \frac{d}{dt}C_{i}=J_{chan}+J_{leak}-J_{pump} \end{array}$$

Additionally, the variable, *h*, for the gating process for inactivation of the IP${}_{3}$ channels is described by:

$$\begin{array}{c} \frac{d}{dt}h=a\left(C_{i}+d_{inh}\right)\left(\frac{d_{inh}}{C_{i}+d_{inh}}-h\right) \end{array}$$

This second differential equation was also simplified by Li and Rinzel and represents the inactivation of the IP${}_{3}$ channels by Ca${}^{2+}$, including the two parameters, *a* (s${}^{-1}$) and d${}_{inh}$ (µM). The parameter, *a*, influences the corresponding time scale for the described process, and the parameter, d${}_{inh}$, defines the corresponding dissociation constant. Apart from the cytosolic Ca${}^{2+}$ balance equation and the added expression term for inactivation, the Li–Rinzel model considers a Ca${}^{2+}$ exchange between the cell and the extracellular medium. It is possible to define additional assumptions for the Ca${}^{2+}$ transporters at the plasma membrane. For our further analysis, a small and constant Ca${}^{2+}$ influx into the cell is sufficient. It is defined by the term, J${}_{in}$ (µMs${}^{-1}$), and in the following, we will include it in the cytosolic Ca${}^{2+}$ balance equation:

$$\begin{array}{c} \frac{d}{dt}C_{i}=J_{chan}+J_{leak}-J_{pump}+J_{in} \end{array}$$

Setting J${}_{in}$ to zero corresponds to the situation of an isolated cell.

### 2.3. Summary of Model Modifications

We have modified the original Li and Rinzel model to make it suitable for tobacco cells. The ER is confirmed as an inner Ca${}^{2+}$ store in the Li and Rinzel model. In plants, we regard the vacuole as the main inner Ca${}^{2+}$ store. The Ca${}^{2+}$ channels in the membrane of the vacuole are not well described. Although stimulation by IP${}_{3}$ is reported *in vivo* [30], IP${}_{3}$, receptor genes in the *Arabidopsis thaliana* genome have not been found so far [31]. As the involvement of inositol phosphates is confirmed, an IP${}_{x}$ variable was adopted. The adaptation of other parameters is listed in Table 1. As for the parameter values, we stuck to the animal data in the first place. When values had to be modified, we usually found suitable values for our plant system through optimization procedures. Setting the c${}_{1}$ constant to 0.1, we found that it not only improved our simulation results, it also resembles the corresponding vacuole/cytosol volume ratio in plants [14]. Additionally, the IP${}_{x}$ concentration was altered into a stimulus function, described more precisely in the following.

**Figure 1:**
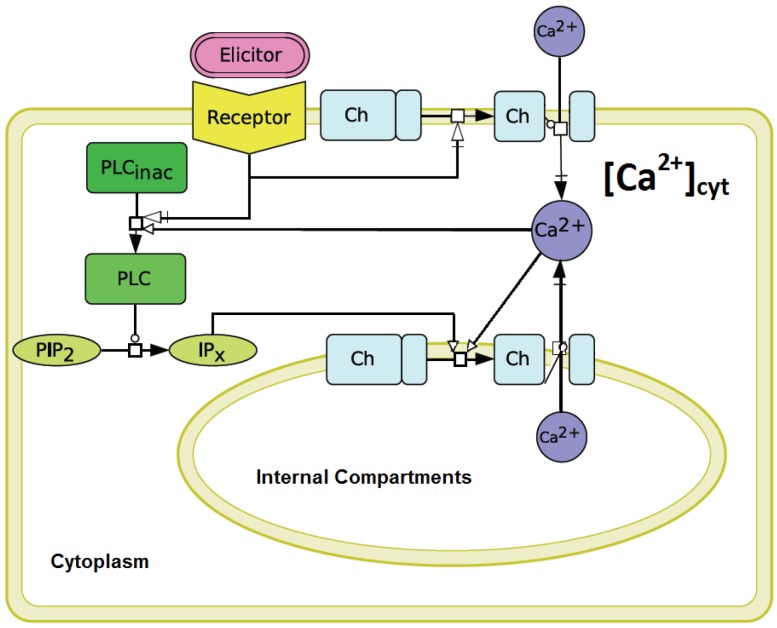
Minimal model for the generation of calcium transients in plant cells upon stimulation by pathogen-associated molecular patterns (PAMPs). Elicitor recognition triggers the influx of Ca${}^{2+}$ ions from outside the cell (illustrated as the transition from a closed to an open channel in the figure) and the production of inositol phosphates (IP${}_{x}$) by phospholipase C activity, which are responsible for the release of Ca${}^{2+}$ ions from inner stores into the cytosol. The molecular identity of, e.g., IP${}_{3}$-activated channels at the membranes of the inner compartments in plants has not been confirmed so far [21]. (Analogously, the Li and Rinzel model considers the activation of the cytosolic Ca${}^{2+}$ release via IP${}_{3}$ and Ca${}^{2+}$. The activation process through Ca${}^{2+}$ is defined as calcium-induced calcium release (CICR).) This figure was made with CellDesigner [33].

**Figure A1:**
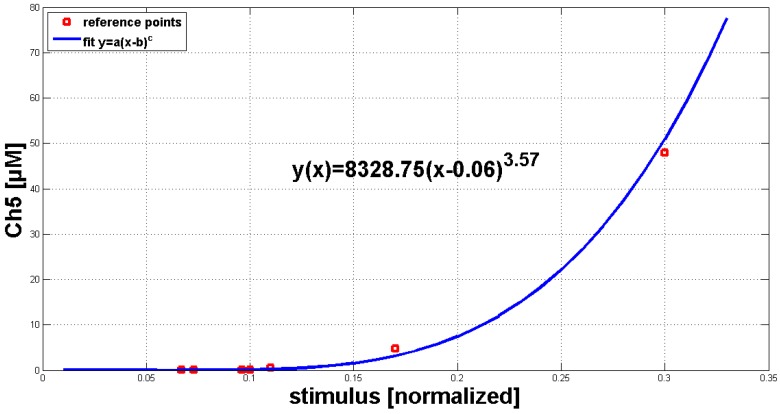
Transfer function that converts normalized values into Ch5 values.

The IP${}_{x}$ concentration variable was modified in order to use it as a stimulus function related to the elicitation in the experiments. This theoretical stimulation is directly effective at the entrance of the inner Ca${}^{2+}$ store. The part of signal transduction between elicitor recognition and the activation of Ca${}^{2+}$ release in the plant system (Figure 1) still needs to be described. To overcome this “black box”, we have, firstly, chosen to define normalized values, between 0 and 1, for our simulated stimulus. Secondly, a transfer function had to be installed to convert the normalized values into the elicitor concentration (Ch5) values used in the experiments. We identified [0-1]-Ch5 value pairs corresponding to the identical cytosolic Ca${}^{2+}$ release, both in the simulations, as well as in the experiments. These value pairs were further used as reference points to build a transfer function converting the normalized [0-1] values of the theoretical stimulus into the Ch5 values of the elicitation applied in the experiments. The use of a normalized stimulus function has the advantage that it can be easily adapted to different elicitors as used in experiments. The signal’s way from the receptor of a plant cell to the entrance of the inner Ca${}^{2+}$ store can then be described by a transfer function being specific for this elicitor. For our elicitations with penta-*N*-acetylchitopentaose oligosaccharides (Ch5), the function y(x) = 8328.75(x − 0.06)${}^{3.57}$ (x represents a normalized value), being the result of a fitting process performed with MATLAB [32], served our purposes well (see Figure A1 in the Appendix).

### 2.4. Model Simulation

Differential equations were solved with MATLAB [32] using the ode45 solver, based on an explicit Runge–Kutta formula.

## 3. Results and Discussion

### 3.1. Cytosolic Calcium Increase upon Elicitation by Penta-N-acetylchitopentaose Oligosaccharides Measured in Tobacco Plant Cell Cultures

**Figure 2:**
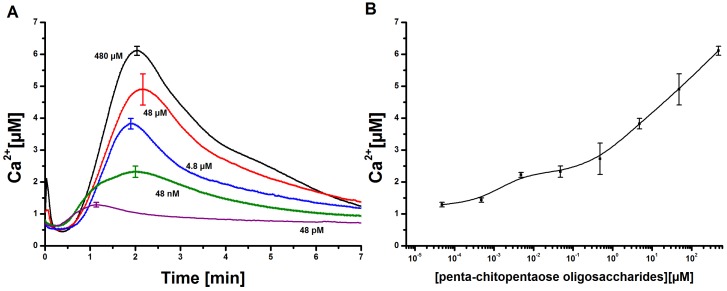
Measurement and quantification of cytosolic Ca${}^{2+}$ concentrations in tobacco cell cultures upon elicitation with *N*-acetylchitopentaose oligosaccharides. (**A**) Dose-dependent Ca${}^{2+}$ kinetics for the acetylchitopentaose oligosaccharides (Ch5) elicitor measured in tobacco plant cell cultures. The elicitor concentration was chosen in the range of 48 pM to 480 µM. Each cytosolic Ca${}^{2+}$ curve has a delay time of 50 s and a faster increase than decrease. Three replicates of every single concentration were taken. The cytosolic Ca${}^{2+}$ curves shown constitute the calculated mean of the three replicates. Error bars indicate standard deviation. (**B**) Dose-response diagram of the measured Ch5-induced Ca${}^{2+}$ transients. The maxima of cytosolic Ca${}^{2+}$ responses are recorded for each concentration. The resulting curve shows a clear rise; higher elicitor concentrations lead to stronger Ca${}^{2+}$ responses. Error bars indicate standard deviation.

In order to directly compare elicitor-induced Ca${}^{2+}$ transients in tobacco cell cultures and the mathematical simulation of these experiments, the effect of penta-*N*-acetylchitopentaose oligosaccharides (Ch5) was first analyzed *in vivo*. Aequorin-transformed tobacco cell cultures were treated with selected elicitor concentrations in the range of 48 pM to 480 µM, and the Ca${}^{2+}$ transients were recorded (Figure 2). The maximum of every single Ch5-induced Ca${}^{2+}$ curve was reached in about one to 2 min after elicitation, depending on the elicitor concentration. The overall duration of the signal was about 7 min, while the delay time took about 50 s, independently of the elicitor concentration. The signal curve is asymmetric. The observed increase of the Ca${}^{2+}$ curve was always faster than the decrease, an important feature considered for the mathematical model. Related cytosolic Ca${}^{2+}$ elevations could be observed with transgenic soybean cells treated with chitotetraose oligosaccharides (Ch4). Fast responses, which peaked within 2–2.5 min, could be detected, and the monophasic behavior of the Ca${}^{2+}$ signal was monitored [12].

### 3.2. Qualitative and Quantitative Simulations of Cytosolic Calcium Signals Induced by Penta-N-acetylchitopentaose Oligosaccharides

It was intended to establish a mathematical model in order to simulate the Ca${}^{2+}$ signature measured in elicitor-treated cell cultures. Special emphasis was put on the simulation of the asymmetric shape of the “on”- *versus* “off”-kinetics. Additionally, the simulation should show the adequate intensity of the Ca${}^{2+}$ response, influenced by the corresponding stimulus activation. To this end, our modified version of the Li and Rinzel model underwent various simulations with different sets of parameters to find matching values that could reproduce a consistent simulation result for a Ch5-induced Ca${}^{2+}$ response in a plant system. In comparison to the parameter-set of the original Li and Rinzel model, the following parameters were observed to be most sensitive: the IP${}_{3}$ permeability (p${}_{IP_{3}}$), the dissociation constant, d${}_{inh}$, and the time scale parameter, *a*, both involved in the Ca${}^{2+}$ inactivation of the channels, the dissociation constant, d${}_{act}$ (Ca${}^{2+}$ activation of the channels), and the total Ca${}^{2+}$ concentration, C${}_{0}$. C${}_{0}$ was calculated by C${}_{ER}$ = [C${}_{0}$− C${}_{i}$]/c${}_{1}$. The constant, c${}_{1}$, is defined by the ER/cytosol volume ratio in the Li and Rinzel model. In order to achieve the appropriate store size in plants, we used the vacuole/cytosol volume ratio of 0.1 [14]. All utilized parameters are listed in Table 1.

**Figure 3:**
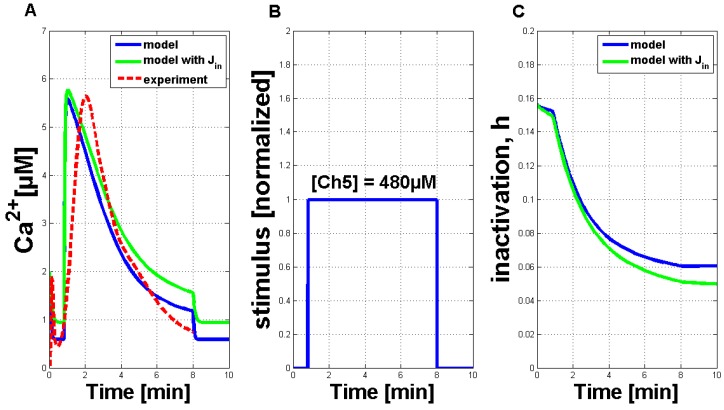
Qualitative comparison of measured Ca${}^{2+}$ transients and simulations of Ca${}^{2+}$ transients with the adapted Li and Rinzel model. (**A**) Comparison of measured cytosolic Ca${}^{2+}$ concentration in tobacco plant cells treated with 480 µM of Ch5 (dashed red line) and simulation with modified values for adapted parameters (solid blue curve), as listed in Table 1 (without the J${}_{in}$ flux). A second simulation (solid green line) is shown under the same circumstances, additionally containing a constant influx, J${}_{in}$, through the plasma membrane. (**B**) IP${}_{x}$ stimulus used for the activation of the system. The value of one of the normalized input corresponds to a Ch5 concentration of 480 µM. (**C**) The inactivation process of the channels in the membrane of the internal store in the plant cell (in analogy to the IP${}_{3}$- and Ca${}^{2+}$-activated channels in the membrane of the ER in the Li and Rinzel model).

**Figure A2:**
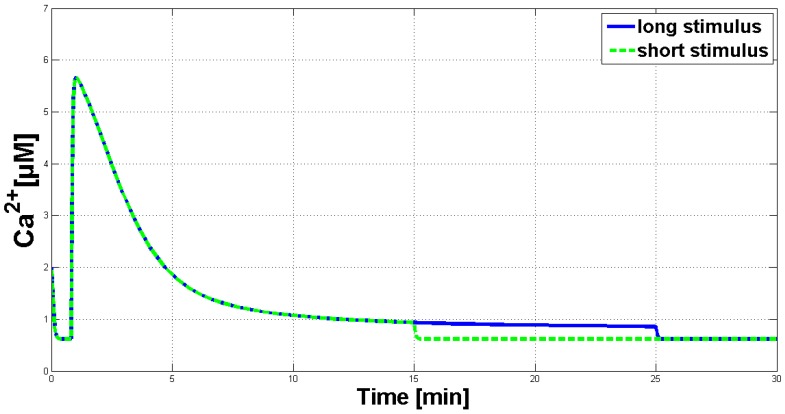
Comparison of simulated Ca${}^{2+}$ transients with the adapted Li and Rinzel model.

The parameters, p${}_{IP_{3}}$ and C${}_{0}$, turned out to be essential for achieving a cytosolic Ca${}^{2+}$ concentration of 6 µM. Both parameters had to be increased to get appropriate Ca${}^{2+}$ responses in the plant system compared to the observed Ca${}^{2+}$ oscillations in pituitary gonadotrophs [16]. The modified parameters, *a* and d${}_{act}$, were important to trigger a single Ca${}^{2+}$ response, due to their influence on the channel kinetics of the IP${}_{3}$ receptor channels in the membrane of the ER in analogy to the gating processes in the membrane of an internal store in a plant cell. Their decrease slows down the two gating processes involved in the Ca${}^{2+}$ interaction. The parameter, d${}_{inh}$, played an important role regarding the refractory behavior of the measured Ca${}^{2+}$ transients and will be discussed later on. In summary, simulations using the parameters given in Table 1 resulted in a qualitative and quantitative output very comparable to the measurements in plant cell cultures. An exemplary simulation result for a Ch5 concentration of 480 µM (solid blue curve) is shown in Figure 3A. It is compared favorably to a single measured Ca${}^{2+}$ signature (dashed red curve). Both curves showed the typical asymmetric structure of the Ca${}^{2+}$ response, the simulation curve’s increase was a little bit stronger. Additionally, a second simulation result (solid green line), including the external influx of Ca${}^{2+}$ ions (defined as J${}_{in}$) is shown in this figure. In comparison to the simulation result without the J${}_{in}$ flux, the general behavior is not strongly influenced. In plant cells, the elicitor detection triggers a primary influx of Ca${}^{2+}$ ions from outside the cell. This can also be included in the model by introducing a dependency of the J${}_{in}$ flux. If J${}_{in}$ is then chosen as zero, the other Ca${}^{2+}$ fluxes will not be triggered. For further analysis, the dependency of J${}_{in}$ was not considered, as the Ca${}^{2+}$ simulations with and without the J${}_{in}$ flux do not considerably differ. Figure 3B shows the IP${}_{x}$ stimulus for the simulated activation of the Ca${}^{2+}$ release (see Figure 3A) with a time period of seven minutes. The length of the simulated Ca${}^{2+}$ response can be varied according to stimulus length (see Figure A2 in the Appendix). In addition, Figure 3C shows the inactivation process of the channels in the membrane of the internal store in the plant cell (in analogy to the IP${}_{3}$- and Ca${}^{2+}$-activated channels in the membrane of the ER).

**Table 1:** List of parameters for the Li and Rinzel model (left), the adapted parameters for the Ca${}^{2+}$ simulations in a plant system induced by Ch5 (middle) and the used values for the simulations in arbitrary units (right).

L-RParameter (Unit)	Adapted parameter	Value
$\upsilon_{1}$→ p${}_{IP_{3}}$ (s${}^{-1}$)	p${}_{IP_{x}}$	140
$\upsilon_{2}$→ p${}_{L}$ (s${}^{-1}$)	p${}_{L}$	0.05
$\upsilon_{3}$→$\upsilon_{ER}$ (µMs${}^{-1}$)	$\upsilon_{store}$	0.5
$k_{er}$ (µM)	$k_{store}$	0.4
$d_{ip3}$ (µM)	$d_{ipx}$	0.1
$d_{act}$ (µM)	$d_{act}$	0.02
$d_{inh}$ (µM)	$d_{inh}$	0.04
*a* (s${}^{-1}$)	*a*	0.001
$IP_{3}$ (µM)	$IP_{x}$	stimulus
$C_{0}$ (µM)	$C_{0}$	7.5
$c_{1}$	$c_{1}$	0.1
$J_{in}$ (µMs${}^{-1}$)	$J_{in}$	0.1
Initial $C_{i}$ (µM)	$c_{i0}$	2
Initial h	$h_{0}$	0.1562

**Figure 4:**
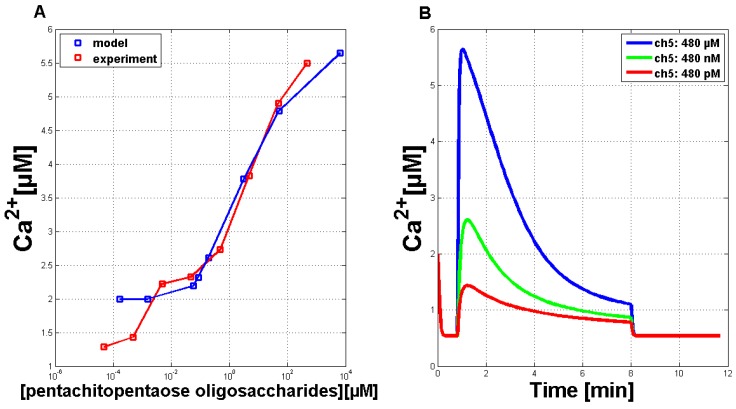
Quantitative comparison of measured Ca${}^{2+}$ transients and simulations of Ca${}^{2+}$ transients with the adapted Li and Rinzel model. (**A**) Comparison of experimental (red curve) and simulated (blue curve) dose-response relationship for the Ch5 elicitor. (**B**) Simulations of single Ca${}^{2+}$ curves in answer to treatment with reduced stimulus activation, in analogy to the reduced Ch5 concentrations as used in the experiments.

In the next step, the model’s dose-response behavior was tested to quantitatively adjust the simulation results. The stimulus used was a normalized function with values between zero and one. With the help of a transfer function (y(x) = 8328.75(x − 0.06)${}^{3.57}$), these values can be converted into the Ch5 concentration as used in the experiments. The maximal concentration of every single Ch5 stimulus is set in relation to the peak of the corresponding Ca${}^{2+}$ answer (Figure 4A, blue line). A theoretical curve similar to the experimental dose-response behavior (Figure 4A, red line) could be achieved. Figure 4B exemplarily shows three Ca${}^{2+}$ simulations treated with reduced stimulus activation. These reduced answers were among those used for the calculation of the dose-response curve given in Figure 4A.

### 3.3. Analysis of Calcium Transients Induced by Penta-N-acetylchitopentaose Oligosaccharides at Two Different Consecutive Time Points

**Figure 5:**
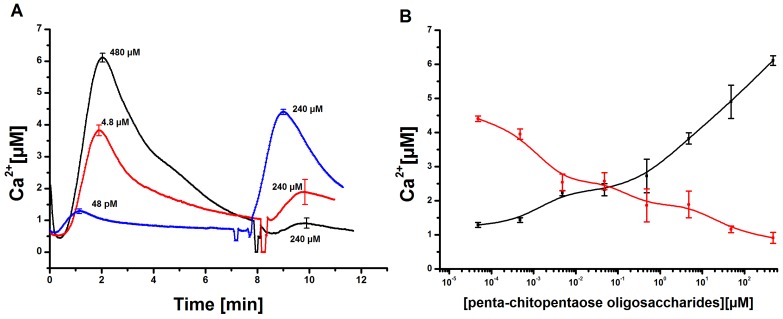
Measurement of cytosolic Ca${}^{2+}$ concentrations in tobacco cell cultures upon elicitation with chitopentaose oligosaccharides at two time points. (**A**) Tobacco cell cultures were elicited with 480 µM, 4.8 µM and 48 pM of Ch5. Approximately 7 min later, a second elicitation with a Ch5 stimulus of 240 µM was carried out. The Ca${}^{2+}$ transients induced by the first and second elicitation were recorded. Error bars indicate standard deviation. (**B**) Dose-response diagram of the first and second response of Ch5 treated Ca${}^{2+}$ signals, shown exemplarily in (**A**). The Ch5 concentration of the first elicitation was reduced (black curve), while the second Ch5 elicitation was constantly held at 240 µM (red curve). Ca${}^{2+}$ answers are always set in relation to the stimulus level of the first stimulation. Error bars indicate standard deviation.

Tobacco cell cultures were stimulated with different concentrations of Ch5 as described before. After about 7 min, the Ca${}^{2+}$ signal almost reached its initial value recorded before stimulation. At this time point, a second elicitation of the already stimulated cell cultures was applied. The second Ch5 concentration was constantly kept at 240 µM. The Ca${}^{2+}$ responses recorded *in vivo* showed the following behavior. In the case of two consecutive strong stimuli of 480 µM and 240 µM of Ch5, the second Ca${}^{2+}$ response was nearly completely suppressed, like already described in published experiments [12,34]. The tobacco cell cultures showed a refractory behavior, *i.e.*, the system was unable to respond to the same stimulus until a certain time had elapsed. In the next experiments, the first Ch5 concentration was gradually reduced, while keeping the second Ch5 concentration constant at 240 µM. With the aid of this approach, it was possible to observe that the first Ca${}^{2+}$ response decreased, while the second Ca${}^{2+}$ response increased. A diagram of the corresponding dose-response relationship shows this in more detail (Figure 5B). Interestingly, the curves show a different behavior. The dose-response curve of the second elicitation shows a moderate decrease, while the dose-response curve covering the first elicitation shows the typical distinct rise.

**Figure 6:**
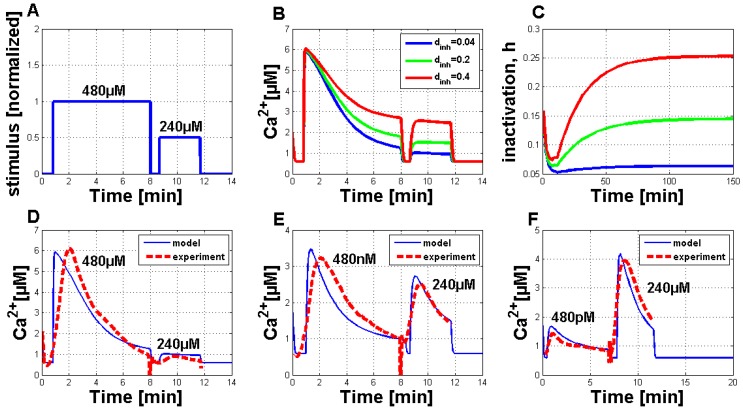
Simulation of Ca${}^{2+}$ signatures with the adapted Li and Rinzel model and qualitative comparison to experimental Ca${}^{2+}$ signatures, focusing on the stimulation at two different time points. (**A**) Normalized IP${}_{x}$ stimulus function for the activation of the simulations in Figure 6B–D. The first elicitation was set to one as a normalized value, and the second elicitation was set to 0.5, related to stimulations with 480 µM and 240 µM of Ch5, respectively. In Figure 6E,F, the stimulus strength was changed. In Figure 6E, the first elicitation was set to 0.11 (corresponding to 480 nM) and in Figure 6F, to 0.06 (480 pM), while the second elicitation remained constant at 0.5 (240 µM). (**B**) Three different Ca${}^{2+}$ simulations of the adapted Li and Rinzel model, activated by the same stimulus function as shown in Figure 6A. Only the parameter variation of d${}_{inh}$ provoked different simulation results. The values of the other parameters were set as listed in Table 1, except $\upsilon_{1}$ = 200 and J${}_{in}$ = 0. (**C**) The corresponding inactivation processes for the Ca${}^{2+}$ simulations in Figure 6B are shown. The parameter set-up was the same as for the simulations in Figure 6B. The time interval of 2.5 h was chosen for a better illustration of process evolution. (**D**) + (**E**) + (**F**) Comparison of experimental (dashed red curve) and simulated (solid blue curve) Ca${}^{2+}$ signatures upon elicitation with two consecutive Ch5 stimuli (6D: 480 µM and 240 µM, 6E: 480 nM and 240 µM and 6F: 480 pM and 240 µM).

Thereafter the analysis of Ca${}^{2+}$ simulations with a modified IP${}_{x}$ stimulus was started. This IP${}_{x}$ stimulus permitted activation of the system at two consecutive time points, in analogy to the experimental setup. This stimulus could be transferred into the corresponding Ch5 concentration of the experiments by using a transfer function, like mentioned before. With the help of a suitable parameter set, it was possible to simulate two consecutive Ch5-induced Ca${}^{2+}$ responses, with the second stimulus directly applied after the end of the first reaction (Figure 6B–F). Before a direct comparison between the experimental observations and the model simulations was possible, a detailed analysis of the Ca${}^{2+}$ simulations, in addition to the simulations for the inactivation process of the channels in the inner compartments of the plant cell, was necessary (Figure 6B,C). For this analysis, Ca${}^{2+}$ simulations with different values for the d${}_{inh}$ parameter were performed (see Figure 6B). It turned out that the d${}_{inh}$ parameter is very sensitive. Increasing its value provokes the second stimulated Ca${}^{2+}$ response, such that it is not suppressed anymore. The corresponding inactivation processes are shown in Figure 6C. With the help of the d${}_{inh}$ parameter sensitivity, it was also possible to find an agreement of experimental and simulated Ca${}^{2+}$ signatures (Figure 6D–F). Figure 6D shows the comparison between the experimental and simulated Ca${}^{2+}$ response elicited at two time points with 480 µM and 240 µM. Furthermore, for the simulated Ca${}^{2+}$ signatures, the second signal is nearly completely suppressed. Figure 6E,F show the simulation results for the elicitation pairs 480 nM–240 µM (Figure 6E) and 480 pM–240 µM (Figure 6F) in comparison to the experimental data. For all simulations, the parameter set-up was chosen as listed in Table 1, except $\upsilon_{1}$ = 200 and J${}_{in}$ = 0. The parameter, d${}_{inh}$, was always set to 0.04.

The aequorin-transformed tobacco suspension cultures reacted to the stimulation by *N*-acetylchitopentaose as already described for soybean cells [9,12]. In comparison to temperature-induced Ca${}^{2+}$ signals [14], a characteristic lag-phase of about 50 s was observed. Obviously, the measured Ca${}^{2+}$ signal is preceded by other signal transduction events, such as protein phosphorylation, other ion fluxes and probably by the activation of phospholipase C [4]. Since the precise structure of the plant signal transduction network triggered by pathogen derived elicitors is not known so far, a reduced model to simulate Ca${}^{2+}$ transients was established. This reduced model is based on the Li and Rinzel model for agonist-induced Ca${}^{2+}$ release in pituitary gonadotrophs [16]. An analogy between the cytosolic Ca${}^{2+}$ release from internal compartments in plant and animal cells was assumed. The Li and Rinzel model [16] considers a Ca${}^{2+}$ release of the ER via IP${}_{3}$, thereby resulting in Ca${}^{2+}$ activation processes leading to cytosolic Ca${}^{2+}$ transients. In the plant cell, we mainly regard the vacuole as the inner Ca${}^{2+}$ compartment. The involvement of inositol phosphates, which are responsible for the cytosolic Ca${}^{2+}$ release after elicitor recognition, is confirmed [24]. Nevertheless, IP${}_{3}$ receptor genes in plants remain to be identified. A physiological role of IP${}_{3}$, or even IP${}_{6}$ in guard cells, could be shown *in vivo* [31].

After the adaptation of the Li and Rinzel model [16], different simulation results were analyzed in detail. Ch5-induced Ca${}^{2+}$ simulations were achieved with modified values for the adopted parameters to make reasonable comparisons with the experiments possible. The simulated Ca${}^{2+}$ curve strongly resembled the experimental Ca${}^{2+}$ signature. The values for the identified parameter-set were not determined *in vivo*, and they are given in arbitrary units. It is a difficult task to identity ligand-gated channels in the vacuolar membrane of plants. A putative two-pore Ca${}^{2+}$ channel, TPC1, in the vacuolar membrane of *Arabidopsis thaliana* has been postulated to be involved in abiotic or biotic stress responses, but it could not be affirmed [35].

Thereafter, the analysis of quantitative simulation results in comparison to the experiments *in vivo* was started. With the normalized IP${}_{x}$ stimulus function, the treatment of varying Ch5 concentrations in the experiments was reproduced, and a typical dose-response behavior could be shown. In the simulations, a transfer function converted the normalized values for the IP${}_{x}$ stimulus into the Ch5 concentrations used in the experiments. The transfer function expresses the early events before cytosolic Ca${}^{2+}$ increase, covering elicitor recognition and the generation of inositol phosphates.

Further on, we focused on the refractory behavior of Ca${}^{2+}$ signals induced by repeated stimulation with Ch5. The repeated stimulation of the same cell culture resulted in significantly reduced Ca${}^{2+}$ transients in response to the second elicitation. To exclude the possibility of osmotic effects, the concentration of the first elicitation was gradually reduced. Most interestingly, the dose-response curves for the first and second elicitation did not sum up to the same value. In other words, a low first elicitation has an over proportional negative effect on the outcome of the second one. Since this outcome is specific for the particular elicitor, the inhibitory effect must target the receptor. A cooperative effect between the receptors or specific modifications of receptor clusters could be a possible explanation [36]. The refractory behavior was integrated into the model by the variable, h, describing the inactivation process of the channels in the membrane of the inner plant Ca${}^{2+}$ compartments. Reasonable simulation results were achieved for a parameter-set with a d${}_{inh}$ parameter of 0.04. A general parameter sensitivity was identified for this parameter. It could be shown that there is a general dependency between the curve shape of h and the corresponding Ca${}^{2+}$ simulation. In the case of a flat curve shape, a second stimulation for the corresponding Ca${}^{2+}$ simulation was not possible. Only if h was allowed to increase, the second stimulation resulted in a second Ca${}^{2+}$ signal.

In general, there are different model approaches for certain plant cell functions. Four major modeling tools to describe different processes in plant cells are exemplarily summarized by Liu *et al.* [37], including differential equations, Boolean networks, network inference and reconstruction and flux balance analysis. The adapted Li and Rinzel model is based on a differential equation approach for Ca${}^{2+}$ transporters, applied to plant cells. Other plant models based on differential equations, including Ca${}^{2+}$ transporters, were developed, e.g., for the generation of action potentials in vascular plants for the cytosolic pool [13], based on the plasmalemma electrical process model by Gradmann [38]. A further differential equation approach for Ca${}^{2+}$ signals, which are triggered by temperature decrease and the exchange of cytosolic and vacuolar pools, was established by Liu and coworkers [14]. They developed a compartment model for ion and growth dynamics in the tip and shank combined in a pollen tube [39]. The major four ion fluxes (Ca${}^{2+}$, K${}^{+}$, H${}^{+}$ and Cl${}^{-}$) are part of the balance equations for the cytosolic and vacuolar pools. The description includes the calcium-induced calcium release (CICR) for the Ca${}^{2+}$ channels in the vacuolar membrane, a buffering component for Ca${}^{2+}$ and H${}^{+}$ ions within the cytosol and the influence of the temperature on the ATP/ADP ratio and the H${}^{+}$ concentration. The values of the parameters are unknown for the different simulations. Anyhow, it was possible to simulate all four ion fluxes and to compare experimental Ca${}^{2+}$ data with the Ca${}^{2+}$ simulations, based on only assumed parameters. Good results were achieved for a rapid temperature decrease.

In summary it can be said that Ca${}^{2+}$ transients induced by mechanical stress [40], temperature or elicitors show some similarities with respect to their shape and absolute concentration of calcium in the cytoplasm. Nevertheless, we can assume that there are marked differences in the underlying signal transduction network, as indicated by the delayed Ca${}^{2+}$ transient that is characteristic for the elicitor perception. For this reason, it could be beneficial to establish different modeling approaches, based on differential equations, as well as other modeling tools, to compare their application to different scenarios. Within this work, a successful adaptation of the Li and Rinzel model to elicitor-induced Ca${}^{2+}$ was carried out. The adapted parameter-set could reproduce Ca${}^{2+}$ simulations, especially focusing on the curve’s shape, dose-response behavior and the activation with two consecutive stimuli.

## 4. Future Perspectives and Conclusions

Pharmacological studies in plant cell cultures have shown that the Ca${}^{2+}$ transients are an essential part of the signal transduction network, allowing the plant to adapt to different environmental situations [41]. Here, the emphasis is set on the network. Other signals have to be transmitted and processed in parallel in order to trigger the specific reaction of the cell [7]. Further studies will clarify the network topology and relations between the network nodes. A still difficult task is to figure out how the dynamics of a calcium transient is transmitted into further signals. Upon the identification and quantitative characterization of further parts of the signal transduction network, more complete models can be established. For the modeling part, the identification of functional modules within the network topology will be of interest, e.g., to find out, whether calcium ions are a hub in the signal transduction machinery of plant defense. Universal functional modules that are not restricted to the description of a specific organism might help to identify general rules. Instead of focusing on specific models based on individual parameters, “scale-free” network models that display general characteristics of information processing systems [42] are an interesting alternative.

## Figures and Tables

This transfer function was used to convert the normalized values (x) of the theoretical stimulus function into the elicitor concentration (Ch5) values (y) used in the experiments.

Shown are two Ca${}^{2+}$ transients, simulated under the same conditions, only stimulus length has been varied. The blue curve shows the reaction to a stimulus being 10 min longer as the input stimulus for the green curve.
